# Neutralization Activity of Standard and Hyperimmune Intravenous Immunoglobulins Against Recently Circulating SARS-CoV-2 Variants

**DOI:** 10.3390/vaccines13070760

**Published:** 2025-07-17

**Authors:** Dongxiao Liu, Lorenza Bellusci, Hana Golding, Surender Khurana

**Affiliations:** Division of Viral Products, Center for Biologics Evaluation and Research (CBER), Food and Drug Administration (FDA), Silver Spring, MD 20993, USA

**Keywords:** COVID-19, SARS-CoV-2, immunoglobulins, convalescent plasma, antibody therapy, treatment, variants

## Abstract

Our study demonstrates that IVIG lots manufactured in 2023–2024 contain neutralizing antibodies against circulating Omicron variants, including KP.3 and XEC. These variants are resistant to all convalescent plasma and IVIG preparations produced prior to 2023. Therefore, recent IVIG lots may provide some protection against COVID-19 caused by circulating SARS-CoV-2 variants.

## 1. Introduction

With an average of 500 deaths per week across the United States during 2024, COVID-19 remains an important public health concern. In the early stages of the SARS-CoV-2 pandemic (2020–2021), post-exposure treatments included several monoclonal antibodies that provided protection from morbidity and lethality. However, rapidly emerging variants of concern (VOCs) in 2023–2024, including JN.1 and its derivatives (KQ.1, KP.3, XEC, etc.), are resistant to all licensed monoclonal antibodies [1,2].

Immunoglobulin products (IGs) administered intravenously (IVIG), manufactured from pooled human plasma, are widely used for the treatment of patients with various immunodeficiency syndromes. In addition, hyperimmune globulins have been developed as prophylaxis or for the treatment of infectious diseases. In response to COVID-19, hyperimmune polyclonal anti-SARS-CoV-2 IVIGs (pi-hCoV-2IG) were manufactured in 2021 from pooled plasma of COVID-19 convalescent patients with SARS-CoV-2 neutralization titers ≥1:320 against the ancestral WA-1 strain. These IVIG products contain IgG at a 10-fold higher concentration than in individual convalescent plasma (CP). Additionally, in 2021, the pooled plasma of individuals (with or without prior SARS-CoV-2 infection) vaccinated against SARS-CoV-2 was used to generate Vx-hCoV-2IG. Since 2022, >90% of the blood donations in the U.S. have had anti-SARS-CoV-2 antibodies, suggesting prior exposure by vaccination, infections, or both (hybrid immunity) [3]. It is therefore expected that the pooled plasma used for the manufacturing of IVIG (thousands of plasmas per lot) will reflect the combined anti-SARS-CoV-2 titers of the plasma donor population.

Our previous study showed that the majority of pi-hCoV2-IG lots and CPs collected after 2023 can neutralize circulating Omicron subvariants (EG.5, HV.1, HK.3, JN.1, JN.4¸ and JD.1.1) at a level (PsVNA 50 titer of >1:40) predicted to provide protection against severe COVID-19 [4]. In contrast, Omicron subvariants BA.2.86, XBB.1.16, XBB.2.3, EG.5, HV.1, HK.3, JN.1, JN.4, and JD.1.1 are more resistant to CP and hCoV-2IG preparations produced prior to 2023. However, the single Vx-hCov-2IG lot made from the plasma of vaccinated individuals that was produced in 2021 not only contains the highest titer against the original WA-1 vaccine strain, but also high neutralization titers against all the prior circulating Omicron subvariants in 2023–2024. Therefore, new lots of IVIG may contain neutralizing antibodies that not only reflect the past exposure history of the plasma donors but also cross-neutralize future emerging subvariants.

The goal of this study was to monitor sequential IVIGs manufactured between 2020 and 2024 for neutralization titers against the newly emerging Omicron variants that have been circulating globally between 2024 and 2025.

## 2. Materials and Methods

### 2.1. Samples and Study Design

IVIG products approved in the United States are polyclonal antibody preparations made from 10,000 or more U.S. plasma donors and may include cold alcohol fractionation (Cohn–Oncley) and anion-exchange and size-exclusion chromatography methods. The final product is sterile-filtered IgG (>95%) and formulated at 100 mg/mL. Twenty intravenous immunoglobulin batches were produced from plasma collected prior to August 2019 (2019-IVIG), and eight IVIG lots made from plasma donations in 2020 (2020-IVIG) and manufactured between October 2020 and January 2021 were obtained from six manufacturers. All plasma units used in the manufacturing of the 2023-IVIG and 2024-IVIG were from US-based collection centers. No COVID-19 exposure (or vaccination history) was collected.

Seventeen pi-hCoV-2IG batches prepared from post-SARS-CoV-2 infection CP collected at least 30 days post-recovery (~200–1000 US plasma donors per lot) were obtained/purchased from four commercial companies for blinded antibody analysis. The plasma units used in the manufacturing of the hCoV-2IG batches were collected in 2020 (during the circulation of ancestral Wuhan, D614G, or Alpha strains) prior to the emergence of the Delta and Omicron VOCs or prior to the availability of COVID-19 vaccines. Nine IVIG lots manufactured in 2023 (2023-IVIG) and seven IVIG lots manufactured in 2024 (2024-IVIG) were obtained from four manufacturers. One hyperimmune intravenous immunoglobulin lot (Vx-hCoV-2IG) produced from plasma collected from SARS-CoV-2 vaccinated individuals at least 2 weeks after their second vaccination (most with prior COVID-19 infection) in 2021 (during the Alpha and Delta circulation) prior to the circulation of Omicron was obtained from one manufacturer.

Seven random CP lots were obtained from recovered COVID-19 patients between May and September 2020 (at least 30 days post-recovery) prior to COVID-19 vaccinations. At the time of collection, SARS-CoV-2 D614G was the predominant strain in the US. Eight CP lots were collected in February 2022 from recovered individuals following Omicron breakthrough infections (probably BA.1), who received at least two doses of COVID-19 mRNA vaccinations.

### 2.2. Neutralization Assay

Samples were evaluated in a qualified SARS-CoV-2 pseudovirion neutralization assay (PsVNA) using the SARS-CoV-2 WA1/2020 strain and eight Omicron subvariants: JN.1.1.1, KP.2, KQ.1, KZ.1.1.1, KP.2.3, KP.3, KP.3.1.1, and XEC circulating in 2024. The mutations in the spike proteins of these Omicron subvariants are shown in Supplementary Table S1. SARS-CoV-2 neutralizing activity measured by PsVNA correlates with PRNT (a plaque reduction neutralization test with an authentic SARS-CoV-2 virus) in previous studies [5,6,7]. However, some antibodies targeting the N-terminal domain of SARS-CoV-2 spike proteins may not show neutralization in the pseudovirus neutralization assay.

Neutralization assays were performed as previously described [5,7]. Briefly, 50 µL of SARS-CoV-2 S pseudovirions (counting ~200,000 relative light units) was pre-incubated with an equal volume of a medium containing serial dilutions (starting at 1:10) of all samples at room temperature for 1 h. Then, 50 µL of virus–antibody mixtures was added to 293T-ACE2-TMPRSS2 cells [10^4^ cells/50 μL; gift from the laboratory of Carol Weiss [5]] in a 96-well plate. The input virus with all SARS-CoV-2 strains was the same (2 × 10^5^ relative light units/50 µL/well). After a 3 h incubation, a fresh medium was added to the wells. Cells were lysed 24 h later, and luciferase activity was measured using the One-Glo luciferase assay system (Promega, Madison, WI, USA). The assay of each sample was performed in duplicate, and the 50% neutralization titer was calculated using Prism 9 (GraphPad Software, San Diego, CA, USA). The limit of detection for the neutralization assay is 1:20. Two independent biological replicate experiments were performed for each sample, and variation in PsVNA50 titers was <10% between replicates.

### 2.3. Quantification and Statistical Analysis

Descriptive statistics were performed to determine the geometric mean titer values and were calculated using GraphPad (Boston, MA, USA, version 9.3.1). All experimental data to compare differences among groups were analyzed using an ordinary one-way ANOVA with Tukey’s pairwise multiple comparison test in GraphPad Prism version 9.3.1. To ensure robustness of the results, absolute measurements were log2-transformed before performing the analysis.

### 2.4. Study Approval and Informed Consent

This study was reviewed and approved by the Food and Drug Administration’s Research Involving Human Subjects Committee (RIHSC #2020-04-02). This study complied with all relevant ethical regulations for work with human participants, and written informed consent was obtained. Samples were collected from adult subjects who provided informed consent to participate in the study. All assays performed fell within the permissible usages detailed in the original informed consent form.

## 3. Results

To determine therapeutic potential against currently circulating SARS-CoV-2 variants, we followed the STROBE reporting guideline for cross-sectional studies. We evaluated neutralization titers of 62 IVIGs and 15 CPs, including pre-pandemic 2019-IVIG (n = 20), 2020-IVIG (n = 8), 2020 convalescent plasma (2020-CP; n = 7), 2022 convalescent plasma (2022-CP; n = 8), post-infection hyperimmunoglobulin IVIG (pi-hCoV-2IG; n = 17), 2023-IVIG (n = 9), 2024-IVIG (n = 7), and 1 post-vaccination hyperimmunoglobulin IVIG (Vx-hCoV-2IG) against the original WA-1 strain and 8 SARS-CoV-2 variants that circulated in 2024. SARS-CoV-2 neutralization assays were performed by using pseudoviruses expressing the spike protein of WA1/2020 or of the Omicron subvariants JN.1.1.1, KP.2, KQ.1, KZ.1.1.1, KP.2.3, KP.3, KP.3.1.1, and XEC (Supplementary Table S1) in 293-ACE2-TMPRSS2 cells as previously described [8,9].

**Figure 1:**
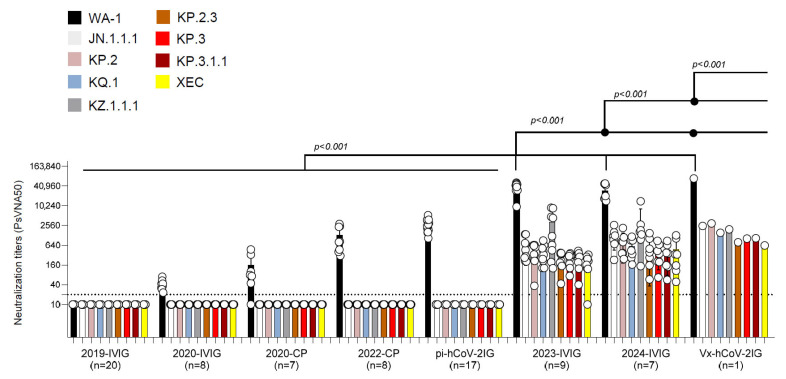
Neutralization activity of IVIG, convalescent plasma, pi-hCoV-2IG, and Vx-hCoV-2IG against SARS-CoV-2 WA1/2020 and circulating Omicron subvariants. Neutralization assays were performed using pseudoviruses expressing the spike protein of WA1/2020 or the Omicron subvariants in 293-ACE2-TMPRSS2 cells. SARS-CoV-2 neutralization titers were determined in each of the pre-pandemic 2019-IVIG (n = 20), 2020-IVIG (n = 8), 2020 convalescent plasma (2020-CP; n = 7), 2022 convalescent plasma (2022-CP; n = 8), post-infection hyperimmunoglobulin IVIG (pi-hCoV-2IG; n = 17), 2023-IVIG (n = 9), 2024-IVIG (n = 7), and post-vaccination hyperimmunoglobulin IVIG (Vx-hCoV-2IG; n = 1) preparations. The assay was performed in duplicate to determine the 50% neutralization titer (PsVNA50). The heights of the bars indicate the geometric mean titers, and the whiskers indicate 95% confidence intervals. The horizontal dashed line indicates the limit of detection for the neutralization assay (PsVNA50 of 20). Differences between SARS-CoV-2 strains were analyzed by an ordinary one-way ANOVA using Tukey’s pairwise multiple comparison test in GraphPad Prism version 9.3.1, and the *p*-values are shown.

Convalescent plasma collected from recovered COVID-19 patients in 2020 (2020-CP) and 2022 (2022-CP), as well as post-infection hyperimmunoglobulin lots (pi-hCoV-2IG), showed high neutralization titers against the WA-1 but demonstrated minimal or no PsVNA titers against the recent Omicron variants (Figure 1 and Supplementary Table S2). The 2019-IVIG lots manufactured before the COVID-19 pandemic contained no SARS-CoV-2 neutralizing antibodies (Figure 1). The 2020-IVIG lots manufactured early in the COVID-19 pandemic contained low titers against WA-1 (PsVNA50: 23–70), and no neutralizing titers against recent Omicron subvariants. The 2023-IVIG and 2024-IVIG lots contain high titers against ancestral WA-1 (PsVNA50: 9555–51,810 and 14,334–48,724, respectively), reflecting the high SARS-CoV-2 seroprevalence in plasma donors and continued back boosting of memory B cells specific for the original SARS-CoV-2 infecting strain. More importantly, these recently manufactured IVIG lots have moderate titers against 2024 circulating variants, even though most of the donors were exposed to earlier Omicron variants (Supplementary Table S2). Surprisingly, the single lot of Vx-hCov-2IG produced in 2021 that contained the highest titer against the original WA-1 strain (PsVNA50: 69,551) also demonstrated high neutralization titers against all the recently circulating Omicron subvariants (PsVNA50 ranging between 627 and 2963) (Figure 1).

## 4. Discussion

Our findings demonstrate that standard IVIG lots manufactured in 2023–2024 contain neutralizing antibodies against recently emerging Omicron VOCs, including KP.2, KP.2.3, KP.3, and XEC, which are resistant to all licensed Mabs. Recently, subvariant LP.8.1 has become dominant globally. However, a report by Chen et al., using convalescent sera after JN.1 and KP.3.3 infection and vaccine sera (JN.1 mRNA), demonstrated no significant differences in neutralization resistance between KP3.1.1., XEC, and LP.8.1 [10]. Plasma collections often occur within 4–6 months of IVIG manufacturing. Therefore, the circulating strains during 2023–2024 (XBB, JN.1, KP.2, and KP.3), in addition to recent booster vaccines (XBB and JN.1-like), most likely contributed to the broad coverage of the recently manufactured (2023–2024) standard IVIGs. Moreover, the PsVNA50 titers of the 2023–2024 manufactured IVIGs are predicted to be protective based on a recent study on high-titer CP [11]. The unexpected breadth of neutralization against recently circulating Omicron strains may also reflect the ongoing affinity maturation of cross-reactive B cells induced by repeat exposures to vaccination/infection, resulting in broader high-affinity antibody repertoires that neutralize emerging SARS-CoV-2 variants [12].

The study limitations include testing of only one Vx-hCoV-2IG lot due to the unavailability of additional lots. Assessments of additional Vx-hCoV-2IG lots would strengthen conclusions about the vaccine-induced antibody breadth of IVIG preparations. Secondly, the clinical benefit of IVIG should be evaluated, including a correlation between PsVNA50 titers with reductions in the severity of COVID-19 or time to symptom resolution, in patients receiving IVIG regularly.

These cross-neutralizing IVIG lots can be an important intervention, particularly for immunocompromised patients such as those with HIV/AIDS or undergoing chemotherapy and patients with various autoimmune and neurological diseases, to prevent or ameliorate the outcome of exposure to circulating and emerging SARS-CoV-2 strains. However, to our knowledge, no new SARS-CoV-2 hyperimmune IVIG lots are under production.

## 5. Conclusions

In conclusion, currently available standard IVIG lots from 2023 to 2024 could reduce the severity of COVID-19 symptoms in patients infected with circulating SARS-CoV-2 variants and prevent long-term complications associated with COVID-19. As SARS-CoV-2 evolves, it will be valuable to measure neutralizing antibodies against emerging variants in newly manufactured IVIG lots, which are broadly used in vulnerable populations.

## Data Availability

All underlying data shown in the manuscript is provided in Supplementary Table S2 of the Supplementary Materials. S.K. had full access to all the data in the study and takes responsibility for the integrity of the data and the accuracy of the data analysis.

## Supplementary Materials

The following supporting information can be downloaded at: https://www.mdpi.com/article/10.3390/vaccines13070760/s1, Table S1: SARS-CoV-2 variants mutations introduced in the spike plasmid for production of SARS-CoV-2 pseudovirions for analysis in PsVNA; Table S2: Neutralization titers of convalescent plasma, IVIG and hCoV-2IG against SARS-CoV-2 variants.
