# Supplementary material for: Neutralization Activity of Standard and Hyperimmune Intravenous Immunoglobulins Against Recently Circulating SARS-CoV-2 Variants
Source: Vaccines (Basel). 2025 Jul 17;13(7):760. doi: 10.3390/vaccines13070760 (PMC12299002; doi:10.3390/vaccines13070760)
Supplement: Supplementary file 1 [file vaccines-13-00760-s001.zip › vaccines-3698774-supplementary.pdf]

Suppplementary Table S1: SARS-CoV-2 variants mutations introduced in the spike plasmid for production of SARS-CoV-2 pseudovirions for analysis in PsVNA.

SARS-CoV-2 variant	Mutations constructed in the spike plasmids*
Omicron (JN.1.1.1)	BA.2.86 mutations (T19I, delL24, delP25, delP26, A27S, G142D, V213G, G252V, G339D, S371F, S373P, S375F, T376A, D405N, R408S, K417N, N440K, S477N, T478K, E484A, Q493R, Q498R, N501Y, Y505H, D614G, H655Y, N679K, P681H, N764K, D796Y, Q954H, N969K, D339H, K356T, A570V, V445H, R493Q, F486P) + L455S +T572I
Omicron (KP.2)	BA.2.86 mutations (T19I, delL24, delP25, delP26, A27S, G142D, V213G, G252V, G339D, S371F, S373P, S375F, T376A, D405N, R408S, K417N, N440K, S477N, T478K, E484A, Q493R, Q498R, N501Y, Y505H, D614G, H655Y, N679K, P681H, N764K, D796Y, Q954H, N969K, D339H, K356T, A570V, V445H, R493Q, F486P) + L455S+ R346T+F456L
Omicron (KQ.1)	BA.2.86 mutations (T19I, delL24, delP25, delP26, A27S, G142D, V213G, G252V, G339D, S371F, S373P, S375F, T376A, D405N, R408S, K417N, N440K, S477N, T478K, E484A, Q493R, Q498R, N501Y, Y505H, D614G, H655Y, N679K, P681H, N764K, D796Y, Q954H, N969K, D339H, K356T, A570V, V445H, R493Q, F486P) + L455S+R346T+T572I
Omicron (KZ.1.1.1)	BA.2.86 mutations (T19I, delL24, delP25, delP26, A27S, G142D, V213G, G252V, G339D, S371F, S373P, S375F, T376A, D405N, R408S, K417N, N440K, S477N, T478K, E484A, Q493R, Q498R, N501Y, Y505H, D614G, H655Y, N679K, P681H, N764K, D796Y, Q954H, N969K, D339H, K356T, A570V, V445H, R493Q, F486P) + L455S+R346T+F456L+T572I
Omicron (KP.2.3)	BA.2.86 mutations (T19I, delL24, delP25, delP26, A27S, G142D, V213G, G252V, G339D, S371F, S373P, S375F, T376A, D405N, R408S, K417N, N440K, S477N, T478K, E484A, Q493R, Q498R, N501Y, Y505H, D614G, H655Y, N679K, P681H, N764K, D796Y, Q954H, N969K, D339H, K356T, A570V, V445H, R493Q, F486P)+ L455S+S31+H146Q+R346T+F456L+V1104L
Omicron (KP.3)	BA.2.86 mutations (T19I, delL24, delP25, delP26, A27S, G142D, V213G, G252V, G339D, S371F, S373P, S375F, T376A, D405N, R408S, K417N, N440K, S477N, T478K, E484A, Q493R, Q498R, N501Y, Y505H, D614G, H655Y, N679K, P681H, N764K, D796Y, Q954H, N969K, D339H, K356T, A570V, V445H, R493Q, F486P) + L455S+F456L+Q493E+V1104L
Omicron (KP.3.1.1)	BA.2.86 mutations (T19I, delL24, delP25, delP26, A27S, G142D, V213G, G252V, G339D, S371F, S373P, S375F, T376A, D405N, R408S, K417N, N440K, S477N, T478K, E484A, Q493R, Q498R, N501Y, Y505H, D614G, H655Y, N679K, P681H, N764K, D796Y, Q954H, N969K, D339H, K356T, A570V, V445H, R493Q, F486P) + L455S + S31
Omicron (XEC)	BA.2.86 mutations (T19I, delL24, delP25, delP26, A27S, G142D, V213G, G252V, G339D, S371F, S373P, S375F, T376A, D405N, R408S, K417N, N440K, S477N, T478K, E484A, Q493R, Q498R, N501Y, Y505H, D614G, H655Y, N679K, P681H, N764K, D796Y, Q954H, N969K, D339H, K356T, A570V, V445H, R493Q, F486P) + L455S+ T22N+F59S+F456L+Q493E+V1104L

* Variant mutations are in relation to the ancestral WA-1 spike sequence.

Supplementary Table S2: Neutralization titers of convalescent plasma, IVIG and hCoV-2IG against SARS-CoV-2 variants*

	WA-1	JN.1.1.1	KP.2	KQ.1	KZ.1.1.1	KP.2.3	KP.3	KP.3.1.1	XEC
**IVIG batches produced in 2019 prior to COVID-19**									
2019-IVIG-1	10	10	10	10	10	10	10	10	10
2019-IVIG-2	10	10	10	10	10	10	10	10	10
2019-IVIG-3	10	10	10	10	10	10	10	10	10
2019-IVIG-4	10	10	10	10	10	10	10	10	10
2019-IVIG-5	10	10	10	10	10	10	10	10	10
2019-IVIG-6	10	10	10	10	10	10	10	10	10
2019-IVIG-7	10	10	10	10	10	10	10	10	10
2019-IVIG-8	10	10	10	10	10	10	10	10	10
2019-IVIG-9	10	10	10	10	10	10	10	10	10
2019-IVIG-10	10	10	10	10	10	10	10	10	10
2019-IVIG-11	10	10	10	10	10	10	10	10	10
2019-IVIG-12	10	10	10	10	10	10	10	10	10
2019-IVIG-13	10	10	10	10	10	10	10	10	10
2019-IVIG-14	10	10	10	10	10	10	10	10	10
2019-IVIG-15	10	10	10	10	10	10	10	10	10
2019-IVIG-16	10	10	10	10	10	10	10	10	10
2019-IVIG-17	10	10	10	10	10	10	10	10	10
2019-IVIG-18	10	10	10	10	10	10	10	10	10
2019-IVIG-19	10	10	10	10	10	10	10	10	10
2019-IVIG-20	10	10	10	10	10	10	10	10	10
**IVIG batches produced in 2020 (circulating SARS-CoV-2 strains: Wuhan, D614G, and Alpha)**									
2020-IVIG-1	36	10	10	10	10	10	10	10	10
2020-IVIG-2	32	10	10	10	10	10	10	10	10
2020-IVIG-3	70	10	10	10	10	10	10	10	10
2020-IVIG-4	43	10	10	10	10	10	10	10	10
2020-IVIG-5	53	10	10	10	10	10	10	10	10
2020-IVIG-6	43	10	10	10	10	10	10	10	10
2020-IVIG-7	29	10	10	10	10	10	10	10	10
2020-IVIG-8	23	10	10	10	10	10	10	10	10
**Convalescent plasma batches produced from COVID-19 survivors collected in 2020 (circulating SARS-CoV-2 strains: Wuhan, D614G, and Alpha)**									
2020-CP-1	333	10	10	10	10	10	10	10	10
2020-CP-2	101	10	10	10	10	10	10	10	10
2020-CP-3	10	10	10	10	10	10	10	10	10
2020-CP-4	79	10	10	10	10	10	10	10	10
2020-CP-5	44	10	10	10	10	10	10	10	10
2020-CP-6	73	10	10	10	10	10	10	10	10
2020-CP-7	473	10	10	10	10	10	10	10	10
**Convalescent plasma batches produced from COVID-19 survivors collected in 2022 (circulating SARS-CoV-2 strains: Omicron BA.1 and BA.2)**									
2022-CP-1	2419	10	10	10	10	10	10	10	10
2022-CP-2	827	10	10	10	10	10	10	10	10
2022-CP-3	307	10	10	10	10	10	10	10	10
2022-CP-4	399	10	10	10	10	10	10	10	10
2022-CP-5	802	10	10	10	10	10	10	10	10
2022-CP-6	2858	10	10	10	10	10	10	10	10
2022-CP-7	2335	10	10	10	10	10	10	10	10
2022-CP-8	469	10	10	10	10	10	10	10	10
**pi-hCoV-2IG batches produced from COVID-19 donors (circulating SARS-CoV-2 strains: Wuhan, D614G, and Alpha)**									
hCoV-2IG-1	3064	10	10	10	10	10	10	10	10
hCoV-2IG-2	2102	10	10	10	10	10	10	10	10
hCoV-2IG-3	3459	10	10	10	10	10	10	10	10
hCoV-2IG-4	1555	10	10	10	10	10	10	10	10
hCoV-2IG-5	1047	10	10	10	10	10	10	10	10
hCoV-2IG-6	2050	10	10	10	10	10	10	10	10
hCoV-2IG-7	1836	10	10	10	10	10	10	10	10

hCoV-2IG-8	2438	10	10	10	10	10	10	10	10
hCoV-2IG-9	2452	10	10	10	10	10	10	10	10
hCoV-2IG-10	2977	10	10	10	10	10	10	10	10
hCoV-2IG-11	3522	10	10	10	10	10	10	10	10
hCoV-2IG-12	2777	10	10	10	10	10	10	10	10
hCoV-2IG-13	1602	10	10	10	10	10	10	10	10
hCoV-2IG-14	3472	10	10	10	10	10	10	10	10
hCoV-2IG-15	3834	10	10	10	10	10	10	10	10
hCoV-2IG-16	5285	10	10	10	10	10	10	10	10
hCoV-2IG-17	3825	10	10	10	10	10	10	10	10
**IVIG batches produced in 2023 (circulating SARS-CoV-2 strains: Omicron XBB.1.5, XBB.1.16, XBB.2.3, EG.5, BA.2.86, JN.1, JN.4, HK.3, HV.1, JD.1.1)**									
2023-IVIG-1	30201	212	37	129	197	43	74	40	10
2023-IVIG-2	42551	204	238	129	647	172	302	251	127
2023-IVIG-3	51810	696	638	637	8912	210	253	332	211
2023-IVIG-4	49281	1372	648	893	4679	337	362	431	330
2023-IVIG-5	45821	462	557	422	4352	348	312	334	241
2023-IVIG-6	44281	293	317	213	447	375	338	361	285
2023-IVIG-7	30452	740	336	327	1203	367	299	282	245
2023-IVIG-8	38161	1373	620	516	8627	188	173	192	161
2023-IVIG-9	9555	266	212	243	127	149	129	124	121
**IVIG batches produced in 2024 (circulating SARS-CoV-2 strains: KP.1, JN.1, JN.1.7)**									
2024-IVIG-1	43925	1189	366	264	1637	268	204	141	106
2024-IVIG-2	16768	931	360	344	2711	155	197	156	154
2024-IVIG-3	47724	702	834	440	1830	387	349	359	337
2024-IVIG-4	15980	228	243	164	147	58	66	56	49
2024-IVIG-5	48724	1566	2145	894	14070	499	431	375	335
2024-IVIG-6	14334	1262	800	894	955	1013	668	647	1036
2024-IVIG-7	20119	2628	1107	1159	1368	1487	864	864	1283
**Vx-hCoV-2IG batches produced from SARS-CoV-2 vaccinated plasma donors (mRNA vaccinated US plasma donors with prior COVID-19 with either Alpha or Delta variants)**									
Vx-hCoV-2IG	69551	2470	2963	1528	1943	783	1021	1043	627

*** PsVNA titer Cut-off value: 1:10.**
